# An Account of the Practice of One of the Physicians of the Westminster General Dispensary, and of the Western Dispensary, from the 20th of August, to the 20th of September

**Published:** 1806-10

**Authors:** S. Fothergill

**Affiliations:** Southampton Street, Strand


### *An Account of the Practice of one of the Physicians of the Westminster General Dispensary, and of the Western Dispensary, from the 20th of August, to the 20th of September.*

| ACUTE DISEASES. | | CHRONIC DISEASES. | |
|---|---|---|---|
| Synochus - - - | 5 | Pulmonary Consumption | 9 |
| Typhus - - - | 3 | Scrophula - - | 2 |
| Acute Rheumatism - | 7 | Cough and Dyspnœa | 15 |
| Peripneumony - - | 2 | Pleurodyne - - | 5 |
| Pleurisy - - - | 1 | Asthma - - - | 1 |
| Measles - - - | 3 | Asthenia - - | 12 |
| Urticaria - - | 1 | Chronic Rheumatism | 9 |
| Erysipelas - - | 1 | Lumbago - - | 3 |
| Cholera - - - | 4 | Cephalalgia and Vertigo | 6 |
| Acute Diseases of Infants | 10 | Tic Douloureux - | 1 |

Epilepsy,

| | | | |
|---|---|---|---|
| Epilepsy - - - | 1 | Syphilis - - - | 2 |
| Paralysis - - | 3 | Sore Throat without Fever | 2 |
| Pyrosis - - - | 2 | Psora - - - | 2 |
| Bilious Vomiting - | 5 | Porrigo - - - | 2 |
| Dyspepsia - - | 3 | Scabies - - - | 1 |
| Colic - - - | 1 | Icthyosis Cruris - | 2 |
| Jaundice - - | 1 | Leucorrhœa - - | 6 |
| Diarrhœa - - - | 14 | Menorrhagia - | 5 |
| Gastrodynia - - | 5 | Chlorosis - - | 3 |
| Worms - - - | 6 | Amenorrhœa - - | 5 |
| Dropsy - - - | 10 | Abortion - - | 2 |
| Gravel and Dysury - | 3 | | |

During the last month, bowel-complaints have been numerous, and still continue to form a considerable portion of the list of diseases. Some cases were severe, though none that I know of terminated fatally. Cholera, one of the most violent complaints which affect the stomach and bowels, is generally so alarming on its first attack, that medical assistance is immediately called in, and though death sometimes takes place in the course of a few hours, the disease is often evidently and quickly relieved by medicine. Most practitioners have some favourite remedy with which they have generally succeeded in curing this complaint; in addition to whatever medicine is given, chicken or veal broth may be taken with singular benefit in copious draughts; and glysters of the same materials, adding laudanum as circumstances may indicate, will frequently diminish the excessive irritation of the bowels.

Some patients have been suddenly seized with giddiness in the head, pain in the stomach, nausea and loss of appetite, accompanied with considerable languor and debility; these symptoms in some cases were followed by bilious vomiting, and in general have been soon relieved by an emetic, two or three doses of calomel and rhubarb, and afterwards bitters. This mode of treatment however, does not always succeed, I have been disappointed in one or two instances recently, and the late Dr. Fothergill has observed that in some cases of bilious vomiting, emetics did not seem to be so beneficial as might have been expected; the pain often returned after their use with increased severity, and the tendency to vomiting was more continual; he found great efficacy in the saline mixture with absorbents and a few drops of thebaic tincture, with a dose of rhubarb. Dr. Huxham, again says, "Wherever an acrimonious and bilious colluvies abounds it will be exceeding proper to discharge it, either by a vomit or purge, purge, since the chief situation of the disorder is in the primæ viæ, the viscera of the abdomen, and the mesaraic vessels."

So much do certain diseases depend upon the variation of weather, that by looking over a list of diseases we might determine with some degree of accuracy, whether the state of the air had been hot or cold, dry or moist. Since the last Report, amongst some very fine days we have been subject to some which were particularly wet and cold, so as to induce many people to have recourse to fires, a very useful precaution on such occasions; for we are much more susceptible of disease from a cold damp day in the decline of summer, when the body has been accustomed to a high degree of temperature. In consequence of these sudden variations, rheumatism, coughs, and pulmonary complaints have been numerous; some cases of fever have also fallen under my care. Typhus fever, of late so little known in London, has again occurred. Of the three patients now reported, two are convalescent, the other continues in a state of extreme danger; one of them lived in a house where a man had died in a fever a month ago.

Of the patients affected with summer fever, one was a female, who previous to her present attack, had been subject to a phthisical complaint. In addition to the symptoms of fever noticed in a former Report, she had great pain at the scrobiculus cordis, expectorated thick yellow mucus copiously, had a teazing cough, and as the fever abated the pulmonary affections seemed to increase; her strength is reduced very low, and though she is now free from fever, and the pain in the chest is removed by the application of a blister, she continues in a state of great danger; the remedies which are usually effectual in restoring the exhausted powers of nature after a fever, seeming to aggravate the distressing cough and complaint of the lungs. In these lamentable cases, where the treatment necessary for one symptom is prejudicial to another, we must attend to that which is likely to be the most fatal, and when life is in danger, not delay applying a powerful remedy, because some particular symptom may be rendered worse.

*Southampton Street, Strand, Sept.* 23, 1806. S. FOTHERGILL.

MEDICAL

